# Surface Transmission or Polarized Egress? Lessons Learned from HTLV Cell-to-Cell Transmission

**DOI:** 10.3390/v2020601

**Published:** 2010-02-10

**Authors:** Jing Jin, Nathan Sherer, Walther Mothes

**Affiliations:** 1 Section of Microbial Pathogenesis, Yale University School of Medicine, 295 Congress Ave., New Haven, CT 06536, USA; 2 Department of Infectious Diseases, King’s College School of Medicine, London SE1 9RT, UK

## Abstract

Commentary on Pais-Correia, A.M.; Sachse, M.; Guadagnini, S.; Robbiati, V.; Lasserre, R.; Gessain, A.; Gout, O.; Alcover, A.; Thoulouze, M.I. Biofilm-like extracellular viral assemblies mediate HTLV-1 cell-to-cell transmission at virological synapses. *Nat. Med.* **2010**, *16*, 83–89.

Many viruses spread more efficiently from cell to cell by direct cell-cell contact rather than by using a cell-free mode. Cell-to-cell spread permits rapid spreading as well as evasion of neutralizing antibodies [1]. The human T cell leukemia virus type 1 (HTLV-1) could well represent the champion among cell-cell contact-dependent viruses. It is poorly infectious as cell-free virus, but efficiently spreads in cultures of lymphocytes [2]. HTLV-1's almost complete dependence on cell-cell contact predicts a rich biology by which cell-cell contact promotes HTLV-1 transmission. In a recent report in Nature Medicine, Pais-Correia, Thoulouze and colleagues propose that HTLV-1 particles move from cell to cell in complex with numerous extracellular matrix (ECM) components, forming large surface associated biofilm-like structures [3].

Using confocal and electron microscopy, Pais-Correia and colleagues observed that HTLV-1 accumulates at the surface of infected cells in a meshwork of virally induced extracellular matrix containing ECM components such as collagen and galectin-3 [3] (Figure 1A). Interestingly, tetherin, identified as an antiviral factor that prevents cell-free release of viruses from infected cells [4], is also part of these large structures and may play a role in the retention of HTLV-1 at the surface of infected cells. The co-localization of these extracellular components with HTLV-1 to large assemblies that can be seen at the surface of infected T cells by scanning electron microscopy is striking and very convincing [3]. Importantly, these structures were detected on uninfected target cells following co-culture with infected cells (Figure 1A). Removing these extracellular viral assemblies mechanically, by extensive pipetting, or by treatment with heparin reduced the efficiency of HTLV-1 spreading. ECM components are known to locally enrich signaling molecules thereby critically influencing cellular signal transduction pathways. ECM components may similarly function to concentrate HTLV-1 virions, thereby enhancing overall infectivity. Furthermore, ECM components are rich in carbohydrates that may serve to shelter released extracellular HTLV particles from immune surveillance. The authors liken these structures to bacterial biofilms rich in bacterially produced polysaccharides.

A surface-based mechanism for HTLV-1 transmission contrasts with a previously proposed model for polarized HTLV-1 assembly and spread at sites of cell-cell contact (Figure 1B *versus* 1A) [5–8]. In 2003, Igakura, Bangham and colleagues observed the accumulation of HTLV-1 Gag, Env and genome at the interface between primary HTLV-1 infected and uninfected T cells and subsequently detected viral transfer to the uninfected cell [5]. The movement of viral components to the cell-cell interface was accompanied by a reorientation of the microtubule organizing center (MTOC) as well as accumulation of the cytoskeletal effector protein talin at the zone of cell-cell contact (Figure 1B). Because of similarities to the accumulation of antigen and the polarization observed in the immunological synapse, the authors called these cell-cell interfaces virological synapses [5]. Subsequent work by the same group demonstrated an important role for the HTLV-1 accessory protein Tax in the up-regulation of ICAM-1, that further contributes to the formation of polarized synapses [7,8]. Electron tomography of cell-cell contact sites revealed HTLV particles released into synaptic clefts between infected and uninfected primary lymphocytes, supporting a model involving polarized virus assembly and transmission across the synapse for efficient transmission (Figure 1B) [6].

While it is interesting that both groups have arrived at different conclusions studying the same virus and using related techniques, we emphasize that mechanisms of surface transmission and polarized assembly may not be mutually exclusive and that both processes will likely contribute to the spread of many viruses, not just HTLV-1. For instance, our group has observed contributions from either pathway to the spreading of the murine leukemia virus (MLV) in fibroblast cells (Figure 1C, D) [9,10]. We recently observed the tendency of nascently assembled MLV virions to remain associated with the surface of producer cells after budding, at least in part due to heparin-sensitive virus-cell interactions with cell surface glycosylaminoglycans (and not unlike interactions observed between HTLV-1 and ECM compoenents). Surface retained viruses were competent for transfer to uninfected target cells during transient episodes of contact between infected and uninfected cells (Figure 1C) [10].

Additionally, we have also observed the establishment of long-term interactions between infected and uninfected target cells, regulated by strong binding of viral Env proteins on the infected cell surface to specific receptor molecules on the uninfected cell surface [11]. For many interactions lasting more than 30 min, virus assembly was preferentially directed to sites of cell-cell contact, a phenomenon requiring that the integrity of the cytosolic tail of Env be intact (Figure 1D) [9]. Assembly at Env-enriched cell-cell contacts was up to 55-fold more prevalent than at other regions of the plasma membrane. Thus, studying MLV cell-to-cell transmission, we have documented cellular mechanisms of lateral, surface-based exchange governed by extracellular moeities as well as polarized assembly and transmission at the cell-cell interface likely regulated by intracellular signaling (Figure 1C, D).

For the human immunodeficiency virus (HIV), evidence for surface transmission was first obtained during the study of the ability of dendritic cells to capture and transfer HIV to T cells, a mechanism mediated by C-type lectins resident at the surface of the dendritic cell [12–15]. Moreover, in striking similarity to HTLV-1, an accumulation of evidence supports polarized assembly and egress at virological synapses formed between infected and uninfected T cells [16,17]. The recent description of polysynapses formed between infected cells and multiple target cells argues against the idea that MTOC reorientation is a broad requirement for polarized virus transmission [18]. However, these observations do reinforce the notion that mechanisms of surface-based transfer and polarized egress both contribute to the efficiency of HIV-1 spread. In general, we predict that in the case of a chronically infected cell, viruses may be sequestered at the surface of infected cells and be passed on at a later time point when cells transiently interact with uninfected cells. In contrast, if cells interact with uninfected target cells early after infection or for prolonged periods of time, virus assembly can be polarized towards the cell-cell interface [9]. Interestingly, the electron tomography study of HTLV-1 spreading performed by Bangham group revealed released particles accumulating both at the periphery of the synapse and within the synaptic cleft for a continuously infected cell line. However, particles from naturally infected CD4+ T cells were found only at the synaptic cleft [6].

In the end, we believe that the work by Pais-Correia, Thoulouze and colleagues will be remembered as an intriguing and stimulating report that sheds light on the role of extracellular matrix components in viral spreading. Given that ∼80% of virus infections enter people at mucosal surfaces [19], the role of the complex ECM is massively understudied. Early work suggests that extracellular components may exhibit enhancing as well as inhibitory roles in viral transmission [20,21]. Clearly, it can be expected that the role of the ECM in virus transmission will become a very fruitful area of study.

## Figures and Tables

**Figure 1.**
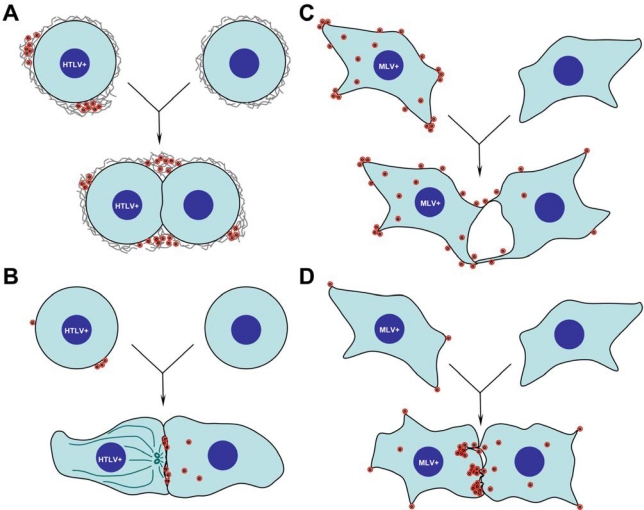
**Models of retrovirus cell-to-cell transmission. A**. HTLV particles released from infected T cells form biofilm like-structure enriched with extracellular matrix components on the cell surface [3]. When infected cells contact uninfected cells, extracellular viral particles spread from cell to cell at the periphery of the viral synapse. **B**. HTLV virus budding is polarized towards the viral synapse [5,6]. Viruses are released into synaptic clefts to infect neighboring T cells. In the infected cells, the MTOC (dark green ovals) is reoriented towards the synapse indicating cell polarity. **C.** Completely budded MLV particles are retained on the cell surface of chronically infected fibroblasts [10]. Upon contact with uninfected cells, particles can spread to uninfected cells via transient fingertip-like cell-cell contacts. **D.** MLV assembly and budding are polarized towards a long-lived and stabilized cell-cell contact zone formed between infected and uninfected fibroblasts [9]. *De novo* assembled viral particles go onto to infect neighboring cells.
